# Intra-peritoneal leiomyoma of the round ligament in a patient with Mayer-Rokitansky-Küster-Hauser (MRKH) syndrome

**Published:** 2016-12

**Authors:** G Salem Wehbe, R Bitar, T Zreik, M Samaha, C Walter, Z Sleiman

**Affiliations:** Lebanese university, Faculty of Medical Sciences; Lebanese American university school of medicine; Geneva university; Lebanese American university school of medicine, the European Academy for Gynecological Surgery.

**Keywords:** Leiomyoma, Mayer-Rokitansky-Küster-Hauser, Middle East, round ligament

## Abstract

**Background:**

The occurrence of an extra-uterine leiomyoma, arising from the intra-peritoneal portion of the round ligament in a lady with Müllerian agenesis diagnosed at the age of forty is extremely rare. We report a case of this rare combination in a Middle Eastern woman.

**Case:**

A 40 years old lady, primarily amenorrheic, presented to our clinic for an infertility consultation. The work- up showed features suggestive of Mayer-Rokitansky-Küster-Hauser (MRKH) syndrome with a leiomyoma arising from the intra-peritoneal part of the round ligament.

## Introduction

The Mayer-Rokitansky-Küster-Hauser (MRKH) syndrome or Müllerian agenesis is characterized by the congenital absence of the upper vagina, cervix and uterus. Its incidence is 1:4,500 female births (Morcel and Camborieux, 2007). It is the second most common cause of primary amenorrhea following ovarian failure (Reindollar et al., 1981). Girls with Müllerian agenesis have normal childhood and they usually do not seek professional advice until puberty when the syndrome presents as primary amenorrhea, otherwise they have normal development of secondary sexual characteristics, normal external genitalia, functional ovaries, and a normal karyotype of 46, XX (Morcel and Camborieux, 2007).

In this case report, we present a case of intra-peritoneal leiomyoma of the round ligament in a patient diagnosed, for the first time at age of forty, to have the Mayer-Rokitansky-Küster-Hauser (MRKH) syndrome.

## Case description

**Fig. 1:**
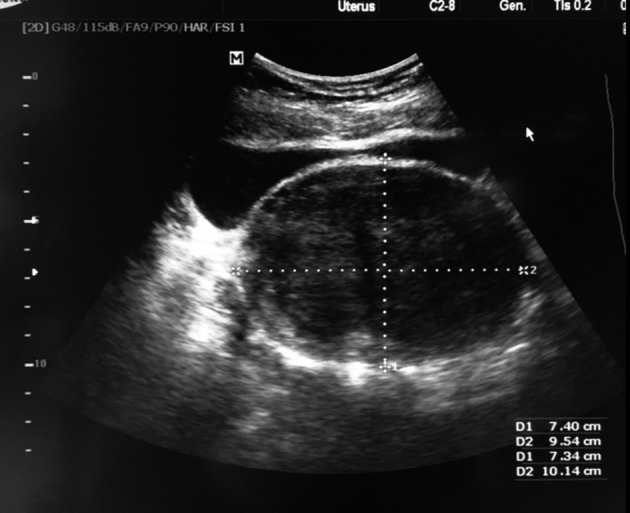


A 40 years old Iraqi lady presented to our private gynaecology clinic 3 months after marriage for infertility problem. Upon primary clinical assessment, she reported primary amenorrhea for which has never been evaluated. Otherwise, her previous medical and surgical histories were normal. Pubarche started at 10 years of age, and thelarche at 12 years. Her family history was negative. Physical examination showed a normal stature lady with normal breasts, axillary and pubic hair development. No hirsutism, acne or galactorrhea. Inspection of genital area showed normal external genitalia and a blind vaginal pouch. The pelvic examination revealed a palpable mobile firm mass. Office tans-abdominal pelvic ultrasound showed 7 x 9 centimetres heterogeneous well-delineated pelvic mass containing many calcifications (Fig. 1).

**Fig. 2:**
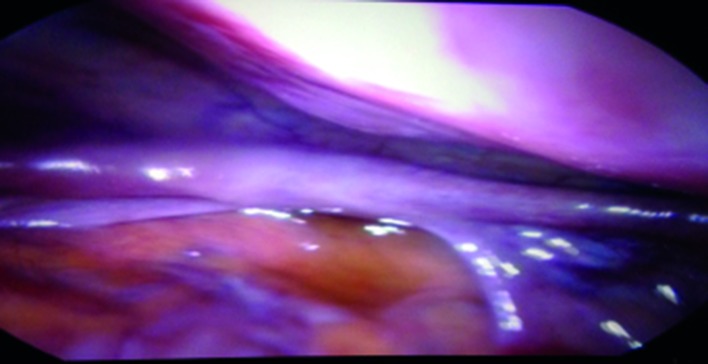
— Laparoscopic view showing the Müllerian agenesis.

**Fig. 3:**
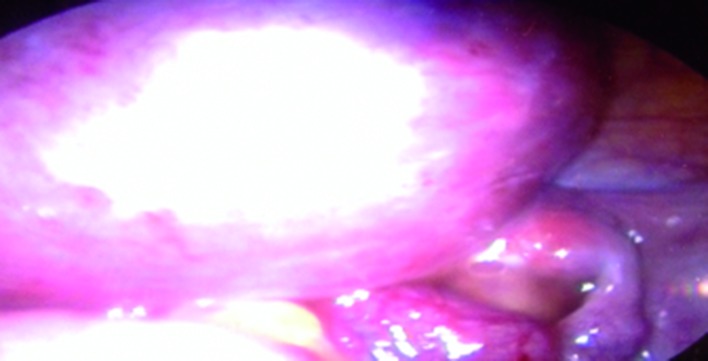
— Laparoscopic view showing the right round ligament leiomyoma

Ovaries were normal, but the uterus couldn’t be clearly identified. Laboratory work-up including full endocrine assessment and tumour markers was normal. Mayer-Rokitanski-Küster-Hauser (MRKH) syndrome was then suspected. An abdominal ultrasound showed both normally located kidneys. A pelvic MRI was suggested in order to prove the diagnosis and characterize the pelvic mass, but the lady refused it because of financial limitation. She asked to skip to a definitive diagnostic procedure. She was scheduled for a diagnostic laparoscopy as being part of primary amenorrhea work up and for the pelvic mass assessment. Diagnostic laparoscopy showed normal ovaries and fallopian tubes, the total absence of the uterus (Fig. 2) and a solid pelvic mass arising from the right-sided intra-peritoneal part of the round ligament (Fig. 3).

In the absence of a written consent of the patient to perform the mass removal, the patient was rescheduled for a laparotomy. In fact, with the doubt of the mass type, and the fear of the presence of leiomyosarcoma, we did not suggest a laparoscopic myomectomy with the use of power morcellator. We counselled the patient concerning the possibility of vaginoplasty and the fertility potential with the help of a surrogate mother. Laparotomy was performed in another clinic one month later and the pathology report revealed a leimyoma with some epithelioid features, moderate myxoid and hyalinized changes.

## Discussion

Leiomyomas arising from Müllerian remnants in patients with Mayer-Rokitansky-Küster-Hauser syndrome are uncommon but should be suspected in women in whom a pelvic mass develops after the confirmation of the Müllerian defect. Authors reported few cases of this rare combination (Fletcher et al., 2012; Farber et al., 1978; Deligeoroglou et al., 2004; Yan and Mok, 2002; Rawat et al., 2013).

Tumours affecting the round ligament are rare. The most commonly found tumours are leiomyomas. Other tumours have been reported such as endometrioma, mesothelial cysts, secondary adenocarcinomas, sarcomas...(Breen and Neubecker, 1962). One-half to two-thirds of round ligament leiomyomas occur in the extra-peritoneal portion of the round ligament (David and Stanley, 1999).

The uterine round ligaments originates from the embryonic female gubernaculum (Acién et al., 2011). Thus, they are in place in Müllerian agenesis. However, the combination of the occurrence of an extra-uterine leiomyoma, arising from the intra- peritoneal portion of the uterine round ligament in a lady with Müllerian agenesis diagnosed at age of forty is extremely rare. Only one case report of leiomyoma arising from the round ligament in a patient with (MRKH) syndrome has been reported. In contrast to our case, the mass was located in the left inguinal canal mimicking an incarcerated inguinal hernia or inguinal adenopathy (Rhee et al., 1999).

In many cultures, infertile ladies suffer discrimination and experience stigmatization and ostracism in their relationships with in-laws and community members (Inhorn, 2003). A woman to be socially acceptable should have at least one biological child (Cui, 2010). In the Middle East, infertility affects the marital dynamics: it leads to instability, divorce or polygamous remarriage (Inhorn, 2003). Thus, it is not surprising to find a woman seeking medical advice for her primary amenorrhea at the age of forty in order to hide her fertility problem, and being diagnosed to have (MRKH) syndrome after her marriage.

Imaging plays a crucial role in the non-invasive and accurate initial diagnosis of (MRKH) syndrome. It is useful in depicting potential associated abnormalities. Radiologists are important elements of the multidisciplinary team taking care of patients with (MRKH) syndrome. Trans-abdominal ultrasonography can initially suggest the diagnosis of Müllerian agenesis, and evaluate associated renal anomalies. The trans-vaginal imaging is often impossible in this group of patients. The three-dimensional (3D) ultrasound is of limited value in (MRKH) syndrome since there are no structures to reformat. MRI is the most accurate technique and is recommended to provide more detailed and objective information. It allows specialists to postpone laparoscopic intervention until constructive surgery is required or when the diagnosis remains doubtful (Rousset et al., 2013).

The role of routine diagnostic laparoscopy as part of the initial work up for patients suspected to have (MRKH) syndrome is of limited value when compared to MRI (Rousset et al., 2013), but still necessary in some situations. Dragusin performed a diagnostic laparoscopy in a case of (MRKH) because imaging couldn’t identify the ovaries. In fact, both ovaries were found in an ectopic high position, in the right and left superior quadrants, with adjacent small fallopian tubes (Dragusin et al., 2014). In our case, it was the presence of a pelvic mass and the non-availability of MRI that lead us to perform the diagnostic laparoscopy.

Laparoscopy allows excellent analysis of a solid pelvic tumour in a patient with (MRKH) syndrome (Lanowska et al., 2009). After adequate characterization of the lesion, laparoscopy offers the surgical access for its removal. Tsin et al. were the first team reporting a successful laparoscopic management of an 8.5 cm size leiomyoma arising from vestigial Müllerian duct in a patient with (MRKH) syndrome (Tsin et al., 2000). We are the first team to report the diagnosis of a large size leiomyoma arising from the intra-peritoneal portion of the round ligament. Because of the older age of the patient (40 years old) and the lack of enough imaging features of the non-malignity of the mass at the time of laparoscopy, the removal was done by laparotomy.

## Conclusion

The presence of leiomyoma is a diagnosis to consider in patients with (MRKH) syndrome. Leiomyoma can originate from the Müllerian remnants but also from the non-Müllerian structures.
